# Influence of Size and Shape Anisotropy on Optical Properties of CdSe Quantum Dots

**DOI:** 10.3390/nano10081589

**Published:** 2020-08-12

**Authors:** Sung Hun Kim, Minh Tan Man, Joong Wook Lee, Kyoung-Duck Park, Hong Seok Lee

**Affiliations:** 1 Department of Physics, Research Institute of Physics and Chemistry, Jeonbuk National University, Jeonju 54896, Korea; kim940122@jbnu.ac.kr; 2 Institute of Theoretical and Applied Research, Duy Tan University, Hanoi 100000, Vietnam; manminhtan@dtu.edu.vn; 3 Faculty of Natural Sciences, Duy Tan University, Da Nang 550000, Vietnam; 4 Department of Physics and Optoelectronics Convergence Research Center, Chonnam National University, Gwangju 61186, Korea; leejujc@chonnam.ac.kr; 5 Department of Physics, Ulsan National Institute of Science and Technology (UNIST), Ulsan 44919, Korea; kdpark@unist.ac.kr

**Keywords:** quantum dots, anisotropy, electronic transition, hidden excitonic feature, level structure

## Abstract

We used low-temperature reactions to synthesize different-sized CdSe quantum dots (QDs) capped with fatty-acid and phosphine ligands. From the correlation of high-resolution transmission electron microscopy and X-ray diffraction (XRD) analyses of the synthesized QDs, we observed size-dependent shape anisotropy. In addition, the recorded XRD patterns revealed mixed crystal facets with zinc blende and wurtzite structures in small-sized QDs. Furthermore, from differential absorption (DA) spectra, we extracted the electronic transition energies for different-sized QDs, which were found to be similar to the calculated values of the quantum size levels associated with band mixing of CdSe QDs with a moderate bandgap. We found that the excitonic absorption peaks are increasingly “hidden” with decreasing QD size because of the crystal structure and crystalline quality. The results show good agreement with the obtained diffraction patterns and the estimation errors obtained from the DA spectra.

## 1. Introduction

Colloidal semiconductor quantum dots (QDs) have attracted considerable interest because of their unique properties, which include quantum confinement effects, large absorption coefficients, tunable and bright emission, and facile control of the spatial distribution of electrons and holes through modulation of the QDs’ size, composition, and shape. Hence, QDs are a promising building block for many potential applications, including light-emitting diodes (LEDs), solar cells, and biomedical imaging [1,2,3,4,5]. In QDs, the size and shape effects strongly influence the quantized electronic energy levels and optical transitions [6,7]. Because these properties are associated with the electronic wave function of localized carriers, spectroscopic measurements and analyses are needed to understand the QD energy levels determined by carrier locations and paths. In the molecular orbitals of single electrons, the highest occupied molecular orbital and lowest unoccupied molecular orbital play an important role in determining the orbital symmetry of CdSe QDs. Specifically, the lowest unoccupied band with Cd 5*s* orbitals and the highest occupied band with Se 4p orbitals show a complicated structure because of the threefold spatial degeneracy.

In CdSe, *j*, as the total angular momentum involved in orbital angular momentum and spin, is a good quantum number because of strong spin–orbit coupling. In the case of the zinc blende and wurtzite structures of CdSe QDs, the crystal field in the wurtzite structure can split into two bands related to the *j* = 3/2 component [8]. This splitting by crystal structure can influence the electron–hole pair state and the growth kinetics, which are decisive factors determining the crystal structure of CdSe QDs.

Crystal facets can be controlled by the crystal growth rates, crystallite size, and the reaction temperature. The *c*-axis separating zinc blende and wurtzite structures leads to the formation of an anisotropic structure such as rod-shaped and hexagonal structures, and this anisotropy limits the range of applications of these QDs. In addition, this anisotropic shape can affect the electronic properties of the CdSe QDs and the degeneracy of their excited hole states [7]. However, the modified zinc blende behavior with respect to the shape anisotropy has scarcely been studied using optical spectroscopy [9]. The shape anisotropy mainly arises from the bond strength of the ligand–monomer complex and its low solubility in solution [10]. Surface–ligand interaction strongly affects the anisotropic effective shape in ligand coverage of the facets [11]. The authors of a previous study reported that elliptic crystallites with oblate, prolate, and ellipsoidal shapes can affect the optical spectra of small-sized CdSe QDs [12]. In particular, small-sized CdSe QDs can generate “hidden” excitonic features such as those observed in the second and third electronic transition states of broadened absorption peaks. In the strong confinement regime, the absorption spectra of CdSe QDs can be theoretically analyzed on the basis of a parabolic approximation [13,14] and the Bohr radius of CdSe is suitable for investigating valence-band degeneracy and energies of quantum size levels, enabling a comparison with electronic transitions assigned on the basis of optical spectra.

In the present work, we investigate the structural and optical properties of CdSe QDs synthesized by the hot-injection technique. High-resolution transmission electron microscopy (HRTEM) and X-ray diffraction (XRD) analysis reveal the size-dependent shape anisotropy of the CdSe QDs. The first through sixth excited states determined from absorption measurements are compared with calculated quantum size levels in *s–* and *p–*symmetry. In particular, featureless absorption peaks of the second and third excited states from the first exciton appear in the differential absorption (DA) spectra. These measurements with quantitative analysis enable the estimate error to be derived from anisotropic properties caused by the crystal field.

## 2. Materials and Methods

### 2.1. Materials

Cadmium oxide powder (CdO, 99.5%), selenium powder (Se, ≥ 99.5%), trioctylphosphine (TOP, tech. grade, 90%), and 1-octadecence (ODE, tech. grade, 90%) were purchased from Sigma–Aldrich (St. Louis, MO, USA). Oleic acid (OA), methanol, acetone, toluene, and chloroform were purchased from Daejung. All chemicals were used as received.

### 2.2. Synthesis of CdSe QDs

For the synthesis of CdSe QDs, 0.0128 g CdO powder (0.1 mmol), 1.58 mL OA, and 20 mL ODE were loaded into a 100 mL three-necked flask. The Cd–OA solution was heated at 80 °C under flowing N_2_ gas and then heated at 120 °C for 1 h under vacuum. After the vacuum heating process, pump/purge cycles were performed at temperatures as high as 250 °C to obtain a clear solution. Se powder (0.0789 g, 0.1 mmol) in 2 mL ODE was then loaded into a 100 mL three-necked flask. TOP (1 mL) was swiftly injected into the stock solution after the solution was subjected to pump/purge cycling to prevent oxidation of the TOP, and the mixture was heated at 120 °C under vacuum. The reaction time of the CdSe solution at 205 °C was controlled, and CdSe QD samples were extracted at 5 s, 1 min, 5 min and 1 h after the injection of the Se–TOP stock solution. The extracted QD samples were precipitated using acetone and methanol. After purification, CdSe QD samples were stored in toluene for further optical characterization.

### 2.3. Characterization

HRTEM images of the CdSe QD samples were recorded with a Cs-corrected TEM (JEM-ARM200F, JEOL) installed at the Center for University-Wide Research Facilities at Jeonbuk National University. For the HRTEM observations, refined CdSe QD samples were dissolved in chloroform and their absorption spectra were recorded to ensure that their concentration was sufficiently low; a carbon-coated copper TEM grid was then dipped into the solution and subsequently dried in air. XRD patterns were recorded using Cu Kα radiation (*λ* = 1.5406 Å). The UV–vis and fluorescence spectra were recorded with a FLAME-S spectrometer (Ocean Optics Inc., Largo, FL, USA).

## 3. Results and Discussion

**Figure 1:**
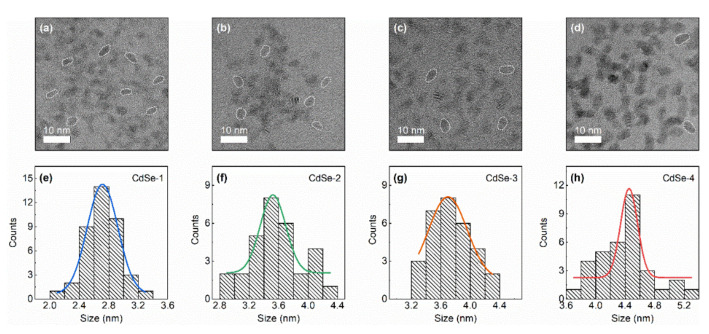
(**a**–**d**) TEM images with shape anisotropy as marked by dotted lines. (**e**–**h**) histograms of size distribution of CdSe QDs with Gaussian-fitting curves (solid line). The average sizes of QDs are (**e**) 2.7, (**f**) 3.5, (**g**) 3.7, and (**h**) 4.5 nm.

The structural morphology, size-dependent optical transitions, and quantum size levels of the CdSe QDs were characterized to elucidate their fundamental properties. The structural morphology observed by TEM confirms that the CdSe QDs exhibit shape anisotropy (Figure 1). The average sizes estimated for samples CdSe–1, CdSe–2, CdSe–3, and CdSe–4 were 2.7 ± 0.5 nm (σ = 20%), 3.5 ± 0.6 nm (σ = 19%), 3.7 ± 0.9 nm (σ = 26%), and 4.5 ± 0.5 nm (σ = 11%), respectively, corresponding to their growth time (5 s, 1 min, 5 min, and 60 min, respectively). The histograms of the particle size distribution for the CdSe QDs were determined from the HRTEM images using the ImageJ software. TEM images of the OA- and TOP-capped CdSe QDs with a small diameter (CdSe–1, CdSe–2, and CdSe–3) show crystalline nanoparticles with shape anisotropy and unclear lattice fringes. These properties emerge at early reaction times because of unstable initial nuclei formation, and this effect is gradually reduced in the back-reaction because of the reconstruction process of the Ostwald ripening effect [15,16]. The narrow size distribution of the QDs (CdSe–4) is attributed to the longer reaction time and the low initial concentration of OA ligand in the ODE noncoordinating solvent [17].

**Figure 2:**
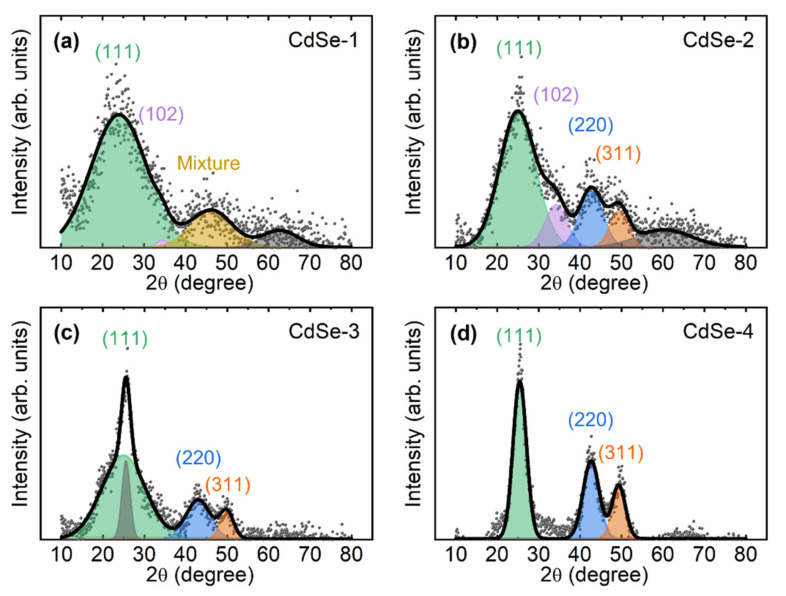
(**a**–**d**) XRD patterns of CdSe QDs: total fitting curve (black solid line) and fitting curves for each XRD peak (color-filled lines) corresponding to a crystal facet.

Figure 2 shows the XRD patterns for CdSe QDs with different sizes. The positions of the XRD peaks are similar to those in the pattern of bulk-phase zinc blende CdSe (largely sized). For small-diameter QDs, the XRD peaks are broad and their intensity decreases with decreasing QD size. The diffraction peaks are determined to correspond to the (111), (220), and (311) planes of the zinc blende CdSe crystalline phase, and an additional peak corresponding to the (400) plane is confirmed at a diffraction angle of 2θ = 62° [18]. The three main distinguished XRD peaks include one peak at a diffraction angle of 2θ = 25° attributed to the (111) crystal facet and two broad peaks at 2θ = 42° and 50° corresponding to the (220) and (311) planes. Additional diffraction peaks in the patterns of the small-sized CdSe QDs (CdSe–1, CdSe–2, and CdSe–3) are confirmed as (102) and (103) wurtzite crystal facets [9]. The existence of the (102) crystal facet at 2θ = 35° and the (103) crystal facet at 2θ = 42° provides conclusive evidence that the small-sized CdSe QDs have a mixed structure. These additional diffraction peaks disappear as the QD size increases. The high crystallinity of the CdSe QDs and their transformation from a mixed structure to the zinc blende structure are possibly caused by a reconstruction of the structure as a result of the longer reaction time. Reaction temperature is also an important factor affecting the crystal structure of CdSe QDs. The low reaction temperature of 205 °C could induce zinc blende stacking faults in the small-sized CdSe QDs capped with fatty acid [10]. In a previous study, the addition of phosphonic acid to TOP–Se resulted in high-quality zinc blende CdSe QDs [19]. A deep valley between the peaks of the (111) and (220) crystal planes and a high intensity of the main diffraction peak have been shown to indicate the formation of a high-quality zinc blende structure [20,21]. In addition, small CdSe QDs have a high surface-area-to-volume ratio, which accelerates oxidation of the QD surface [21]. As a result, the main diffraction peak is broadened and exhibits low intensity.

**Figure 3:**
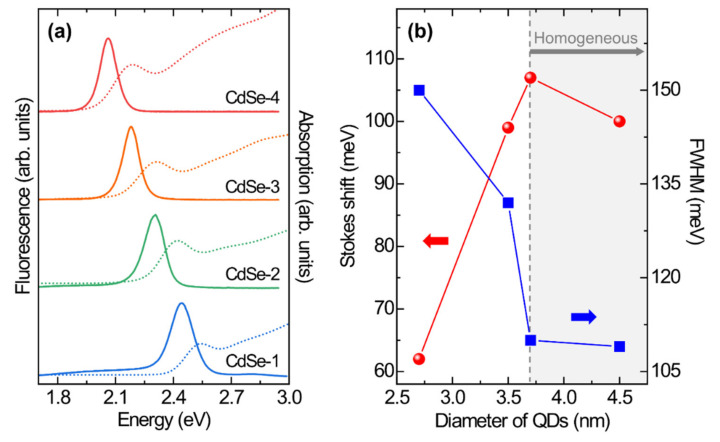
(**a**) The absorption spectra (dashed lines) and fluorescence spectra (solid lines) of CdSe QDs with different sizes. (**b**) The Stokes shifts and FWHMs of CdSe QDs with different sizes.

Figure 3a shows the absorption and fluorescence spectra of OA- and TOP-capped CdSe QDs with particle sizes of 2.5, 3.5, 3.7, and 4.5 nm. With increasing size of the CdSe QDs, the first excitonic transition energy of the absorption peaks (2.54, 2.41, 2.31, and 2.18 eV) and the energy of the fluorescence peaks (2.44, 2.30, 2.18, and 2.06 eV) decrease because of the reduced quantum confinement effect [22]. The full-width at half-maximum (FWHM) of the fluorescence peak of the CdSe QDs, which implies inhomogeneous properties of the CdSe QD ensemble, decreases from 150 to 109 meV with increasing CdSe QD size because of the initial precursor ratio and Ostwald ripening effects [15,17]. The initial Cd:Se precursor ratio influences the maximum peak position, quantum yield, and variation of the FWHM during the growth process [22]. An initial 1:1 precursor ratio leads to a reduction of the FWHM during the growth process as well as to a reduction of the initial FWHM. During growth, the homogeneity of the CdSe QD ensemble is attributable to the role of Ostwald ripening, where the formation of QDs with a critical size leads to homogeneous crystal nucleation and lowering of the surface energy [15].

Stokes shifts of colloidal QDs are related to phonon interactions and polydispersity [23,24]. In turn, the polydispersity of colloidal QDs in solution affects the Stokes shifts [24]. The FWHMs of the first excitonic absorption peak of the 2.5, 3.5, 3.7, and 4.5 nm QDs are 93, 121, 136, and 151 meV, respectively. These results correlate to the Stokes shifts of the colloidal CdSe QDs (Figure 3b). The layer of capping agent on the surface of the CdSe QDs strongly affects their optical properties. The layer of organic ligands in the ODE noncoordinating solvent at the interface of the CdSe QDs leads to mutually separated nanoparticles and their homogeneous growth. A binary ligand system with a Z-type ligand (Cd-oleate) and an L-type ligand (TOP) provides stable surfaces of CdSe QDs, and OA also increases light harvesting [16,25,26]. Therefore, an adjacent exciton transition can be easily distinguished in the absorption spectrum of a material with a large absorption coefficient by differential absorption (DA) spectroscopy, which is a mathematical method for extracting the transition energy obscured by spectral overlap [27].

**Figure 4:**
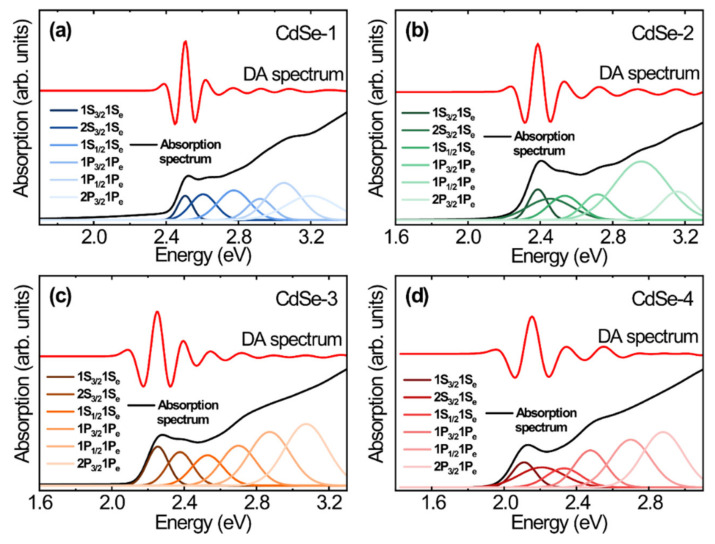
Absorption (black) and differential absorption spectra (red) of (**a**) CdSe–1, (**b**) CdSe–2, (**c**) CdSe–3, and (**d**) CdSe–4 with first through sixth excitonic transitions extracted from differential absorption (DA) spectroscopy analysis.

Figure 4 shows Gaussian peaks of the first through the sixth excitonic transitions for the CdSe QDs, as obtained using the sixth derivative extracted from the original absorption spectrum and plotted as a function of energy. The position change of the centroid of the spectral peaks demonstrates size-induced red shifts of the electronic transitions with increasing particle size. Each original absorption spectrum can be reconstructed from the sum of the Gaussian peaks [27]. To obtain the defined electronic transition energy as a function of the QD size, we extracted the centroid of the spectral peak in Figure 4. The electron–hole pairs of electronic transitions in the energy spectrum determine the absorption spectrum of the CdSe QDs. The interband transitions from the hole states to the electron states should satisfy the selection rules for orbital transitions [13]. In the case of strong confinement, the quantization energy of electronic transitions is dependent on the order of a^−2^, where a is the radius of the CdSe QDs [14]. Therefore, we can calculate the quantum size levels of holes and electrons in the parabolic approximation. We assigned the electronic transitions by comparing the recorded spectra with the theoretically reconstructed spectra. In addition, a mathematical deconvolution process of the original absorption spectra shows estimated errors for the hidden absorption peaks, and second and third absorption peaks are mainly observed for small-sized QDs. These features remain unexplained, and we demonstrate that the hidden absorption peak is likely related to isotropic/anisotropic shapes and other features [7].

**Figure 5:**
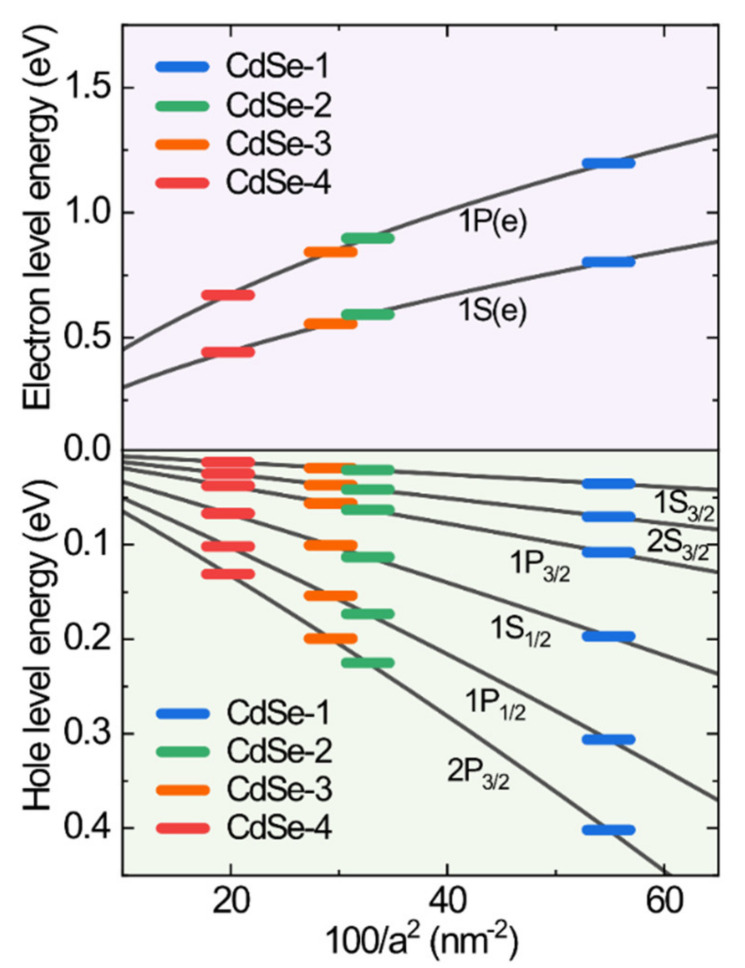
Size-dependent quantum size levels of CdSe QDs. Each solid line indicates a fitting curve of electron and hole levels. Color symbols show electron and hole level energies for CdSe QDs with different sizes.

Figure 5 shows the quantum size level with respect to the dot size for the CdSe QDs. In the hole quantum size levels, we calculated the energy of hole state (ΔE_h_) with s–and p–symmetry [14]. To calculate the size dependence of electron and hole level energies (E_l,k_), we used Equation (1) for the quantum size levels [13]. This expression includes the energy parameters and can explain the role of spin–orbit coupling and the oscillator strength of the CdSe QDs.

(1) $$E_{l,k}=\left[1+2f+\frac{E_{p}}{3}\left(\frac{2}{E_{l,k}+E_{g}}+\frac{1}{E_{l,k}+E_{g}+\Delta }\right)\right]\frac{h^{2}\alpha_{l,k}^{2}}{8m_{0}a^{2}\pi^{2}}$$

where *f* is the parameter of higher bands to the electron effective mass, *E_p_* is the energetic band parameter, and Δ is the spin–orbit coupling of CdSe QDs. We obtained the following set of energy band parameters for CdSe: *f* = −0.42, *E_p_* = 17.5 eV, *E_g_* = 1.74 eV, and Δ = 0.42 eV. The expression *h*^2^*α*_l,k_^2^/8*m*_0_*a*^2^ represents the quantum confinement energy in the strong confinement situation, where h is Planck’s constant, *α_l,k_* is the nth zero of the spherical Bessel function (*l* and *k* are the orbital and principal quantum numbers, respectively), *m*_0_ is the electron effective mass, and a is the radius of the CdSe QDs. In this framework, we simply obtain the nth electronic transition energy of the absorption spectrum. We determined the bulk energy gap of CdSe plus the energy of the hole and electron states. The Coulomb interaction energy is a negligibly small value in the electronic transition. The fitting data for the exciton energies of our QD samples with sizes ranging from 2.7 to 4.5 nm are shown in Figure 5. With increasing size of the CdSe QDs, the observed exciton energies of the first through the sixth excited states, which are transitions to the 1S_e_, 1P_e_ electron levels, decrease. Notably, the observed exciton energies of the first through sixth electronic transition energies excited in the DA spectra are 2.1–3.2 eV, and those calculated for the first through sixth electronic transition energies in the level structure model are 2.1–3.3 eV. From Figure 4 and Figure 5, we can determine the orbital state of the interband transition and compare it with the experimental and theoretical results of the first through the sixth electronic transitions. The quantum size level energies of electronic transitions agree well with the energies of interband transitions obtained by DA spectroscopy.

## 4. Conclusions

We investigated strategies for elucidating electronic transitions of nanocrystals in the strong confinement regime. HRTEM and XRD measurements show an average size of the synthesized CdSe QDs ranging from 2.7 to 4.5 nm and their shape anisotropy. Some QDs exhibit a partially isotropic shape, but most show anisotropic shapes. The hidden excitonic feature is less pronounced in large-sized QDs because of the greater crystalline quality and narrow size distribution. As the size of the CdSe QDs increases, the observed energy of the first through sixth electronic transitions between electron states (1S_e_ and 1P_e_) and hole state (*s*– and *p–*symmetry) decreases. Our experimental results concerning the structural and optical properties of different-sized QDs were quantitatively analyzed using a theoretical model obtained by the quantum size level equation using the parabolic approximation. This approach demonstrates a systematic protocol to adjust excitonic hidden transitions and determine the physical properties of CdSe QDs, as well as to control growth kinetics through manipulation of the surface states and capping agents.
